# Necessity of Tendoachilles Tenotomy in Patients With Congenital Talipes Equinovarus (CTEV) Treated Using the Ponseti Technique: A Prospective Cohort Study

**DOI:** 10.7759/cureus.75324

**Published:** 2024-12-08

**Authors:** Asif Afridi, Bilal Ahmad, Hassaan Ahmed

**Affiliations:** 1 Trauma and Orthopedics, Hayatabad Medical Complex, Peshawar, PAK; 2 Trauma and Orthopedics, Queen Elizabeth Hospital Birmingham, Birmingham, GBR; 3 Orthopedics, Queen Elizabeth Hospital Birmingham, Birmingham, GBR

**Keywords:** clubfoot, congenital talipes equinovarus, foot deformity, pediatric orthopedics, ponsti casting

## Abstract

Aim: This study aims to determine how often Achilles tenotomy is performed on patients who have congenital talipes equinovarus (CTEV) and have been managed with Ponseti serial casts.

Materials and methods: This prospective cohort study took place from November 2021 to May 2023 in the orthopedic unit of Hayatabad Medical Complex, Peshawar, Pakistan. About 38 pediatric patients with CTEV, who received treatment in the form of Ponseti casting, were enrolled in the study. Clinicodemographic data of the patients were recorded. Informed consents were taken prior to enrollment.

Results: Using the Ponseti casting method, 56 feet in 38 pediatric patients with CTEV, were treated. Of 38 patients, 27 (71.05%) were male and 11 (28.94%) were female. Fourteen (36.84%) out of 38 had a right foot, 6 (15.7%) had a left foot, and 18 (47.36%) had bilateral foot involvement. The mean age was 8.8±2.5 weeks (ranging from 5 to 13 weeks) when they were subjected to Achilles tenotomy. The initial mean Pirani score was 3±0.74 while the final Pirani score was 1±0.74 after Ponseti casting, showing the efficacy of Ponseti casting correcting the cavus, varus, and adduction components of clubfoot. The mean number of casts was 5±0.84 to correct the aforementioned components of clubfoot. In 34 feet (60.71%) the residual equines deformity was persistent and Achilles tenotomy was done to make the foot plantigrade. In the end, all 38 patients had plantigrade feet.

Conclusion: Equinus deformity was present in over 60% of the patients following Ponseti serial casting, highlighting the need for tendoachilles tenotomy to make the foot plantigrade. A combination of Ponseti casting and tendoachilles tenotomy resulted in plantigrade feet in 100% of patients.

## Introduction

With a frequency of 1 to 2 per 1,000 live births, congenital talipes equinovarus (CTEV) is highly prevalent in birth abnormalities [1]. With a ratio of 2-2.5:1, male children are afflicted more frequently than female children and nearly half of the cases feature bilateral involvement. CTEV (clubfoot) management has evolved since the time of Hippocrates (400 BC), Guerin (1836), Kite (1964), and Puttipalli (1972) [2,3]. These days, the Ponseti approach, which combines serial casting and weekly manipulation, is the benchmark for treating idiopathic clubfoot [4,5]. An essential part of the Ponseti approach, percutaneous Achilles tenotomy is typically needed to address chronic equinus deformity after forefoot and midfoot deformity repair [6]. Percutaneous Achilles tenotomy occurs 60-90% of the time [3,7,8], with a Ponseti method success rate of 95% [4,6,8]. Tenotomy can be done as an outpatient treatment while under local anesthesia [9].

Our study's goal was to ascertain how frequently Achilles tenotomies are performed on individuals receiving Ponseti serial casting therapy for CTEV.

## Materials and methods

This prospective cohort study was conducted in the orthopedic unit of Hayatabad Medical Complex, Peshawar, Pakistan. This study was approved by the Ethical Review Board of Hayatabad Medical Complex (approval number: 2031). This study took place from November 2021 to May 2023. About 38 patients with idiopathic CTEV were recruited in this study. Before enrollment into the study, a head-to-toe examination was undertaken to exclude any syndrome associated with CTEV. Clinicodemographic (age, gender, laterality, family history, mode of delivery, and Pirani score) data of the patients was noted. The inclusion criteria set were patients with idiopathic clubfoot, age ≤2 years, and patients who agreed to consent to participation in the study. The exclusion criteria were patients with cerebral palsy, arthrogryposis multiplex congenita, neuropathic clubfoot, myelomeningocele, and those with a previous history of treatment and surgery for clubfoot. The Pirani scoring system was used to grade the severity of clubfoot. As a standard treatment modality for CTEV, Ponseti serial casting was conducted weekly. Those patients who achieved plantigrade foot with Ponseti casting and had no dorsiflexion limitation (equines deformity) were prescribed Dennis Brown (DB) shoes, while those patients who had equines deformity at the end of casting were further treated surgically with Achilles tenotomy under local anesthesia. The final cast in dorsiflexion was applied after Achilles tenotomy. Patients were followed regularly on a weekly basis.

## Results

Using the Ponseti casting method, 56 feet in 38 pediatric patients with CTEV were treated. Of 38 patients, 27 (71.05%) were male and 11 (28.94%) were female. About 14 (36.84%) out of 38 had right foot involvement, 6 (15.7%) had left foot involvement, and 18 (47.36%) had bilateral foot involvement. Eight (21.05%) out of 38 patients had a positive family history of clubfoot. Of 38 patients, 7 (18.42%) were preterm, 29 (76.31%) were term, and 2 (5.26%) were post-term delivered. The mean age was 8.8±2.5 weeks (ranging from 5 to 13 weeks) when they were subjected to Achilles tenotomy. The initial mean Pirani score was 3±0.74, while the final Pirani score was 1±0.74 after Ponseti casting, showing the efficacy of Ponseti casting in correcting the cavus, varus, and adduction components of clubfoot. The mean number of casts was 5±0.84 to correct the aforementioned components of clubfoot. In 26 (68.42%) patients and 34 feet (60.71%), the residual equinus deformity was persistent, and Achilles tenotomy was done to make the foot plantigrade. Of these 26 patients, 10 were from bilateral and 6 were from unilateral involvement groups. In the end, all 38 patients had plantigrade feet (Table 1).

**Table 1:** Percentage and frequencies of different variables

Variables	Frequency	Percentage
Gender		
Male	27	71.05%
Female	11	28.94%
Laterality of involved foot		
Right	14	36.84%
Left	6	15.7%
Bilateral	18	47.36%
Need for tenotomy		
Yes	26	68.42%
No	12	31.57%
Gestational age		
Preterm	7	18.42%
Term	29	76.31%
Post-term	2	5.26%
Positive family history of clubfoot	8	21.05%

## Discussion

In our study, the incidence of Achilles tenotomy was 68.42%. The need for tendoachilles tenotomy in children with idiopathic clubfoot was found to be higher when the initial Pirani score was higher [10]. David [11] found that the odds of requiring a tenotomy increased by more than fourfold for every unit increase in the Pirani score. Nevertheless, the necessity for a tenotomy was not linked to a negative outcome. In various studies, it was found that clubfoot is more common in male babies than female [12-14]. The tendon reestablishes continuity between the stumps as soon as three weeks after surgery, with the ability to transmit force to the heel [15,16]. Jain et al. [17] conducted research on the Ponseti technique and compared two groups of residents: one group that had received the proper training in Ponseti casting and tenotomy and the other group that had not received any formal training. When comparing the outcomes of clubfeet managed by these two groups, he observed a substantially higher frequency (p=0.03) of tenotomies in the group that received formal training (73.3% vs. 51.5%). He summed up that the heightened frequency of tendoachilles tenotomies was a result of the trained group's increased confidence in conducting the procedure following the training. Sharma et al. [18] determined that a higher initial Pirani score correlates with a higher number of casts and likely necessitates Achilles tenotomy; thus, parents of affected children should be advised that treatment would be extended and involve a small surgical procedure. We would persuade surgeons who do not support Achilles tenotomy, are reluctant to execute it, or mistakenly think that the equinus of CTEV is rectified when it is not, by precisely calculating the frequency of Achilles tenotomy in our community. Moreover, avoiding an Achilles tenotomy shouldn't be motivated by a fear of problems from the procedure or the unavailability of anesthesia. The limitation of the study was the small sample size and descriptive nature of the study.

Our study did not record any problems associated with percutaneous tenotomy. Research has indicated vascular injuries leading to hemorrhage, pseudoaneurysm, nerve damage causing neurological deficits, and partial tenotomy [19].

## Conclusions

The need for tendoachilles tenotomy following Ponseti serial casting was elevated; equinus deformity was present in >60% of the patients, requiring tendoachilles tenotomy. Parents of the patients should be counseled not to take the need for tenotomy as a failure of Ponseti serial casting. A combination of Ponseti serial casting and timely tendoachilles tenotomy yielded a 100% success rate, making the feet plantigrade.

## References

[1] Joseph EA, Innocent EA, Chukwuemeka A (2016). Descriptive epidemiology and predisposing factors to idiopathic talipes equinovarus in South South Nigeria. J Public Health Epidemiol.

[2] Jaqueto PA, Martins GS, Mennucci FS, Bittar CK, Zabeu JL (2016). Functional and clinical results achieved in congenital clubfoot patients treated by Ponseti's technique. Rev Bras Ortop.

[3] Ponseti IV, Smoley EN (2009). The classic: congenital club foot: the results of treatment. Clin Orthop Relat Res.

[4] Sinha A, Mehtani A, Sud A, Vijay V, Kumar N, Prakash J (2016). Evaluation of Ponseti method in neglected clubfoot. Indian J Orthop.

[5] Hernigou P, Gravina N, Potage D, Dubory A (2017). History of club-foot treatment; part II: tenotomy in the nineteenth century. Int Orthop.

[6] MacNeille R, Hennrikus W, Stapinski B, Leonard G (2016). A mini-open technique for Achilles tenotomy in infants with clubfoot. J Child Orthop.

[7] Anisi C, Asuquo J, Abang I (2018). Frequency of percutaneous achilles tenotomy in the treatment of idiopathic clubfoot using the ponseti method. Niger J Med.

[8] Parikh K, Moradiya NP (2019). Outcomes of Congenital Talipes Equinovarus Treated with Ponseti Method. Int J Contemp Med Res.

[9] Lebel E, Karasik M, Bernstein-Weyel M, Mishukov Y, Peyser A (2012). Achilles tenotomy as an office procedure: safety and efficacy as part of the Ponseti serial casting protocol for clubfoot. J Pediatr Orthop.

[10] Dyer PJ, Davis N (2006). The role of the Pirani scoring system in the management of club foot by the Ponseti method. J Bone Joint Surg Br.

[11] David BH, Olayinka O A, Oluwadare E, Ayodele OE, Joseph O M, Olujide A (2017). Predictive value of Pirani scoring system for tenotomy in the management of idiopathic clubfoot. J Orthop Surg (Hong Kong).

[12] Kruse LM, Dobbs MB, Gurnett CA (2008). Polygenic threshold model with sex dimorphism in clubfoot inheritance: the Carter effect. J Bone Joint Surg Am.

[13] Zionts LE, Jew MH, Ebramzadeh E, Sangiorgio SN (2017). The influence of sex and laterality on clubfoot severity. J Pediatr Orthop.

[14] Salvatori G, Bettuzzi C, Abati CN, Cucca G, Zanardi A, Lampasi M (2020). The influence of laterality, sex and family history on clubfoot severity. J Child Orthop.

[15] Maranho DA, Nogueira-Barbosa MH, Simão MN, Volpon JB (2009). Ultrasonographic evaluation of Achilles tendon repair after percutaneous sectioning for the correction of congenital clubfoot residual equinus. J Pediatr Orthop.

[16] Agarwal A, Qureshi NA, Kumar P, Garg A, Gupta N (2012). Ultrasonographic evaluation of Achilles tendons in clubfeet before and after percutaneous tenotomy. J Orthop Surg (Hong Kong).

[17] Jain G, Lenka S, Chandra M, Mohanty T (2020). Higher rates of Achilles tenotomy after a didactic session on Ponseti method. Arch Bone Jt Surg.

[18] Sharma S, Butt MF, Singh M, Sharma S (2013). The posterior to anterior controlled technique of percutaneous Achilles tenotomy in the correction of idiopathic clubfoot: a technical report. J Pediatr Orthop B.

[19] Dobbs MB, Gordon JE, Walton T, Schoenecker PL (2004). Bleeding complications following percutaneous tendoachilles tenotomy in the treatment of clubfoot deformity. J Pediatr Orthop.

